# Editorial: New technologies and statistical models applied to sports and exercise science research: methodological, technical and practical considerations

**DOI:** 10.3389/fspor.2023.1267548

**Published:** 2023-08-18

**Authors:** Daniel Rojas-Valverde, Miguel A. Gómez-Ruano, Sergio J. Ibañez, Pantelis T. Nikolaidis

**Affiliations:** ^1^ Centro de Investigación y Diagnóstico en Salud y Deporte (CIDISAD-NARS), Escuela Ciencias del Movimiento Humano y Calidad de Vida, Universidad Nacional, Heredia, Costa Rica; ^2^ Sport Injury Clinic, Escuela Ciencias del Movimiento Humano y Calidad de Vida, Universidad Nacional, Heredia, Costa Rica; ^3^ Facultad de Ciencias de la Actividad Física y el Deporte, Universidad Politécnica de Madrid, Madrid, Spain; ^4^ Grupo de Optimización del Entrenamiento y Rendimiento Deportivo, Universidad de Extremadura, Cáceres, Spain; ^5^ School of Health and Caring Sciences, University of West Attica Athens, Athens, Greece

**Keywords:** wearable, statistics, exercice, sport, analysis

**Editorial on the Research Topic** New technologies and statistical models applied to sports and exercise science research: methodological, technical and practical considerations

With new technology and sophisticated statistical models, sports and exercise research has seen a tremendous revolution in recent years. These ground-breaking technologies have altered how researchers approach data collecting, processing, and interpretation by offering deeper insights into numerous elements of human performance, training, and injury prevention (1, 2). This special issue examined the methodological, technical, and practical aspects involved in using these advanced tools and statistical models in sports and exercise science studies.

Technology developments have significantly influenced the study of sports and exercise. Researchers today have an unmatched capacity to acquire and evaluate data in real-time and in ecologically realistic settings because of wearable technology, sensor technologies, virtual reality, and machine learning algorithms (3–5). In addition to improving the precision and accuracy of data gathering, these technologies have created new opportunities for researching intricate physiological, biomechanical, and psychological phenomena in exercise and sport. By adding these technologies to their research processes, scientists may acquire thorough and objective measurements, leading to more reliable discoveries and useful applications (1, 2).

However, using these new technologies has its own unique set of methodological difficulties. The proper choice and positioning of sensors, the verification and dependability of measurements, and the fusion of many data streams are all important considerations for researchers. Additionally, they must solve problems with data management, data processing, and result interpretation. Insights and best practices were provided for researchers looking to incorporate new technology into their studies in this special issue, which investigated these methodological issues. Another essential component of this special issue is modern statistical models (e.g., exploratory factor analysis, machine learning) (4, 6).

Although conventional statistical methods have been widely employed in sports and exercise research, recent advances in statistical modeling have created new data analysis methods. Using models like machine learning algorithms, hierarchical modeling, and network analysis, data from sports and exercise may reveal complex relationships, patterns, and interactions. By incorporating these models into their work, researchers might uncover previously unrecognized insights, identify predictive traits, and enhance decision-making (7, 8).

However, employing these statistical models calls for expertise and close attention to several technical details. Problems with data preparation, model choice, model validation, model interpretation, and result communication must be addressed. Academics would be able to discuss the technical challenges they face when utilizing complicated statistical models in this special issue, as well as highlight successful applications and practical solutions (9–11).

In addition to methodological and technical issues, this special issue explored the practical implications of incorporating new technologies and statistical models into sports and exercise science research. The goal of this kind of research is to translate findings into recommendations that aid players, coaches, and practitioners (12). To effectively use the information gained from these new approaches in a variety of disciplines, including talent identification, injury prevention, and training program design, this special issue accomplished just that (13–15). By bridging the gap between theory and practice, researchers may advance sports and exercise science and encourage systems for making decisions based on evidence.

This special issue set out to investigate the revolutionary effects of these cutting-edge tools in advancing our understanding of human performance, training, and injury prevention. By addressing methodological, technical, and practical challenges, this collection of essays provided researchers useful insights and approaches for incorporating new technologies and statistical models into their investigations. The research presented in this special issue may potentially revolutionize the study of sports and exercise science and promote initiatives to improve health and performance in people.

**Figure 1:**
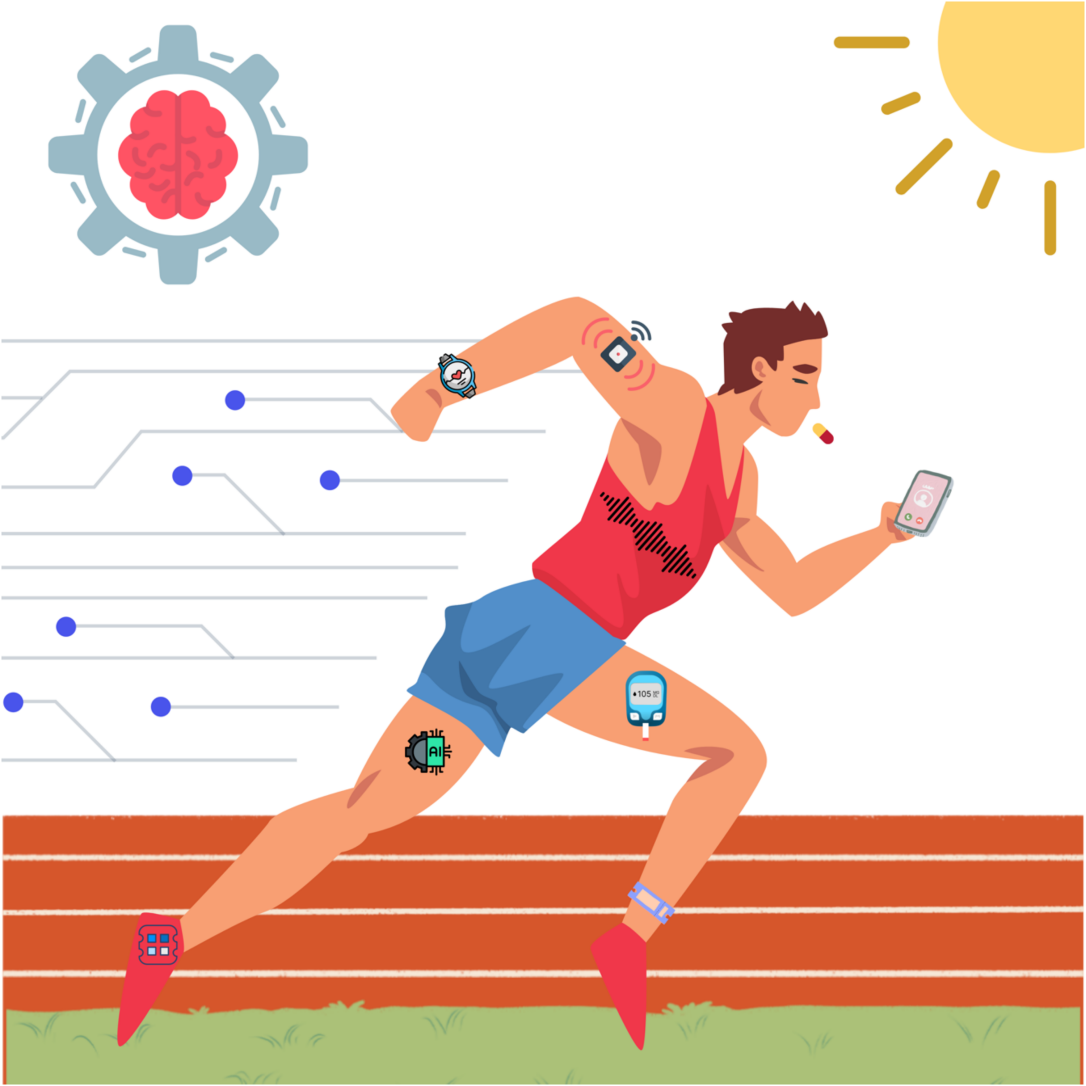
Illustration of new wearable technologies, data generation and statistical models in sports and exercise science research.

This special issue encompasses several articles exploring integrating new technologies and statistical models in sports and exercise science research (see Figure 1). The first article presents a SWOT analysis of smart patches, highlighting their advantages in lightweight comfort, wireless communication, and low cost while acknowledging weaknesses such as low battery capacity (16). The second article compares velocity-based resistance training to percentage-based resistance training, finding that the former yields favorable improvements in power-related parameters (17). The third article examines the association between external load and metabolic profiles in professional football players, emphasizing the need for separate analyses based on sex (18). Lastly, the fourth article proposes a non-linear model for estimating oxygen uptake using wearable devices, showcasing its accuracy in various activity scenarios (19). The fifth study investigates the feasibility of drone-based position detection and its potential applications in performance analysis (20). These articles collectively contribute to advancing sports and exercise science research and its practical implementation by addressing methodological, technical, and practical considerations.

The papers that were written about this topic often emphasize various methodological, technical, and practical issues that need to be considered when incorporating new technology and statistical models into sports and exercise science research. Insights regarding the advantages, drawbacks, and possible applications of smart patches, velocity-based resistance training, metabolic profiles, and drone-based position tracking are gained through analysis. Researchers may improve data collecting, processing, and interpretation by considering these factors, which will inevitably result in improvements to sports and exercise science research and practical applications.
